# Clinical Outcomes of Accelerated Corneal Cross-Linking for Pediatric Keratoconus

**DOI:** 10.1155/2021/1851883

**Published:** 2021-11-18

**Authors:** Abdelrahman Salman, Taym Darwish, Marwan Ghabra, Obeda Kailani, Hussam Khalil, Rafea Shaaban

**Affiliations:** ^1^Honorary Clinical Lecturer at Tishreen University, Scientific Director of Tratous Specialist Eye Center. (Tartus,Syria), P.O. Box: 25, Latakia, Syria; ^2^Head of Department of Ophthalmology, Tishreen University, Latakia, Syria; ^3^Whipps Cross University Hospital, Leytonstone, London, UK; ^4^AKC FRCOphth CertLRS Queen Mary's Hospital, King's College Hospital NHS Foundation Trust, London, UK; ^5^Surgical Eye Hospital, Health Ministry, Damascus, Syria; ^6^Tartous University, Tartus, Syria

## Abstract

**Aim:**

To assess the efficacy and safety of accelerated corneal cross-linking in the treatment of pediatric keratoconus.

**Method:**

In this retrospective case series, 29 eyes of 20 pediatric patients with keratoconus underwent accelerated corneal cross-linking. Treatment was delivered at 10 mW/cm^2^ for 9 minutes with a total dose of 5.4 J/cm^2^. Clinical evaluation included visual acuities and refractive and Scheimpflug corneal tomography assessments. All patients with a minimum follow-up duration of 24 months were included in the study.

**Results:**

Mean ± standard deviation age was 15.41 ± 2.13 years (range: 8 to 18 years). Uncorrected distance visual acuity improved significantly from 0.56 ± 0.28 to 0.42 ± 0.29 logMAR (*P*=0.0003), and corrected distance visual acuity improved significantly from 0.34 ± 0.23 to 0.28 ± 0.22 logMAR (*P*=0.014). The mean manifest refraction spherical equivalent value was significantly reduced (−0.59 ± 0.95 D, *P*=0.0024). While mean flat keratometry and steep keratometry values were not significantly altered (*P* > 0.05 for both), the mean maximum keratometry value was significantly decreased from 56.97 ± 5.24 D preoperatively to 55.84 ± 5.37 D at 24 months postoperatively (*P*=0.003). Maximum keratometry had progressed by >1 D in two eyes (6.89%). Permanent corneal haze was reported in one case (3.44%).

**Conclusion:**

Our 24-month follow-up demonstrated that accelerated corneal cross-linking appears to halt the progression of keratoconus in pediatric patients without apparent complications. Uncorrected and corrected distance visual acuities were also improved.

## 1. Introduction

Keratoconus (KC) is a progressive corneal disease characterized by thinning of the central or paracentral portion of the cornea resulting in irregular astigmatism and visual deterioration [1]. The onset of KC is usually around puberty, although advanced cases have been reported in children as young as 4 years of age [2]. Compared to adults, keratoconus presents a higher rate and faster progression in pediatric patients [3, 4]. The progression of KC may lead to visual impairment in pediatric patients and implications on the social and educational development of the child. Nonsurgical alternative treatments such as spectacles and contact lenses are not always tolerated in children and have a guarded impact on disease progression. Early detection and treatment of keratoconic children may be an effective intervention in the long-term management of KC and ultimately reduce the need for corneal transplantation.

This study aimed to evaluate the visual, refractive, topographic, and tomographic outcomes of the accelerated corneal cross-linking (A-CXL) procedure in pediatric patients with KC.

## 2. Patients and Methods

A retrospective interventional case series study was conducted at the department of ophthalmology, Tishreen University, Syria. All patients included in this study had undergone A-CXL (10 mW/cm^2^ for 9 minutes).

The inclusion criteria consisted of those who were 18 years or younger at the time of surgery with documented KC and with a minimum follow-up duration of at least 24 months after CXL. The diagnosis of KC was established in concordance with the global consensus on keratoconus and corneal ectatic diseases' report [5].

The exclusion criteria included patients with a history of ocular surgery, minimum corneal thickness of less than 330 *μ*m [6], central corneal opacity, and coexisting ocular pathology other than KC or a history of herpetic keratitis. We also excluded KC patients who had clinical findings of vernal keratoconjunctivitis (either typical limbal follicles or tarsal cobblestone papillae at any time point). Contact lens-wearing patients were asked to discontinue wearing their lenses for 3 weeks and 1 week for rigid and soft contact lenses, respectively.

Progression of keratoconus is defined as at least 1 diopter increase in the anterior maximum keratometry or in the manifest refraction spherical equivalent, decrease of 5% in the minimum pachymetry, or loss of at least two lines of the corrected distance visual acuity during the past 12 months [7]. However, based on the Global Consensus on Keratoconus and Ectatic Diseases which states that CXL can be beneficial upon diagnosis in young patients with keratoconus, corneal cross-linking was carried out without waiting for established progression [5].

This study adhered to the tenets of the Declaration of Helsinki and was approved by the Ethics Committee of Tishreen University Hospital, Tishreen University, Syria. Before corneal cross-linking, written informed consent was obtained from at least one parent or legal guardian of each subject.

Clinical evaluation included measurements of uncorrected distance visual acuity (UDVA) and corrected distance visual acuity (CDVA), manifest refraction spherical equivalent (MRSE), slit-lamp biomicroscopy, retinoscopy, and fundoscopy. Topographic, tomographic, and pachymetric data were obtained preoperatively and postoperatively with Scheimpflug/Placido-based corneal tomography, Sirius system (Costruzioni Strumenti Oftalmici, Florence, Italy). The same software version (Phoenix v 2.6) was used preoperatively and postoperatively.

### 2.1. Surgical Procedure

An epithelium-off CXL technique was done in all subjects. Topical anesthesia consisted of proparacaine hydrochloride 0.5% (PROPARACAIN RAMA, Rama Pharma, Pharmaceutical Industry Co., Syria) eye drops administered at 2′minute intervals, starting 10 minutes preoperatively. The central 8-9 mm corneal epithelium was removed using a blunt spatula and dry sponge (without alcohol assistance). Twenty minutes before irradiance, riboflavin with dextran (0.1% riboflavin in 20% dextran; Medio Cross, Germany) solution was applied every 2 minutes. Ultrasonic pachymetry was performed to ensure a minimum corneal thickness of 400 *μ*m prior to ultraviolet A (UVA) exposure. If a corneal thickness of less than 400 *μ*m was observed, one drop of hypoosmolar riboflavin (0.1% in sterile water, Medio Cross hypotonic) was instilled at 10-second intervals over a 2-minute session; this was repeated to swell the thinnest thickness to >400 *μ*m. The 29 eyes were irradiated with the Vega CBM-X Linker (CSO, Italy) using A-CXL 10 mW/cm^2^ UVA for 9 minutes to achieve a total energy of 5.4 J/cm^2^. During the 9 minutes of irradiance, riboflavin solution was applied every 2 minutes. By the end of the procedure, the corneal surface was irrigated with balanced salt solution (BSS), and a soft contact lens was applied for 5 days. Topical moxifloxacin 0.5% eye drop and fluorometholone 0.1% eye drop were prescribed for 1 week and 2 weeks, respectively.

### 2.2. Mean Outcome Measures

Outcomes measured included visual and refractive values, keratometric values (simulated keratometry, flat keratometry (K1), steep keratometry (K2), mean keratometry (K mean), and apex keratometry (K apex)), keratoconus screening indices (keratoconus vertex front (KVf) and keratoconus vertex back (KVb)) which represent the highest point of ectasia on the anterior and posterior corneal surfaces, respectively, the symmetry index of the anterior curvature (SIf), the symmetry index of the posterior curvature (SIb), and total Baiocchi Calossi Versaci index (BCV), and minimum corneal thickness (ThkMin) values.

### 2.3. Statistical Analysis

Data analysis was performed using SPSS version 20 (SPSS, Inc., Chicago, IL) for Windows. Variables were described as a mean and standard deviation (SD). The Wilcoxon rank-sum test was used to compare between preoperative and postoperative values. For the comparison of corneal thickness between hypo- and iso-osmolar groups, the Mann–Whitney *U* test was performed. *P* value <0.05 was considered as significant.

## 3. Results

### 3.1. Demographic Results

A total of 29 eyes of 20 patients with KC were included in this study. Twelve (60%) of the 20 patients were male. The mean ± standard deviation was 15 ± 2.13 years (range: 8 to 17 years).

### 3.2. Visual and Refractive Outcomes

The mean logMAR UDVA and CDVA significantly improved from 0.56 ± 0.28 and 0.34 ± 0.23 preoperatively to 0.42 ± 0.29 and 0.28 ± 0.22 postoperatively, respectively (*P*=0.003 and *P*=0.01, respectively). The mean preoperative MRSE showed a significant improvement from −2.01 ± 1.42 preoperatively to −1.49 ± 1.04 postoperatively (*P*=0.002) (Table 1).

**Figure 1:**
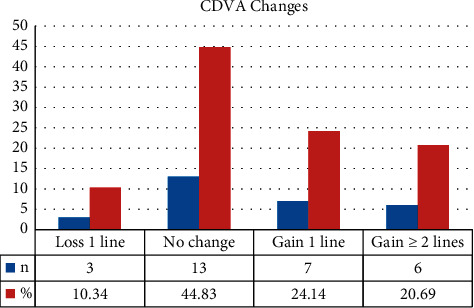
Change in Snellen corrected distance visual acuity (CDVA) following accelerated corneal cross-linking in pediatric patients with keratoconus.

At the last follow-up, Snellen CDVA improved with 13 (44.83%) eyes gaining one line or more and 13 (44.83%) eyes with stable visions and reduced in 3 (10.34%) eyes by one line. No eyes lost more than one line of Snellen CDVA (Figure 1).

### 3.3. Topographic and Pachymetric Outcomes

At the last visit, while all mean simulated keratometric values, K1, K2, and K mean, were not significantly changed (*P* > 0.05 for all), the mean K apex decreased significantly from its preoperative value of 56.97 ± 5.24 D to 55.84 ± 5.37 postoperatively (*P*=0.003). The mean topographic cylinder (Sim cyl) values were not significantly different at the final follow-up compared to baseline values (*P*=0.13).

Mean ThkMin value was not significantly decreased at 2 years postoperatively (7.03 ± 22.24 *μ*m, *P*=0.08). The mean keratometric, topographic, and minimum corneal thickness values are shown (Table 2).

Table 3 demonstrates minimum corneal thickness changes between hypoosmolar and iso-osmolar riboflavin groups. At 24 months after CXL, the reduction in mean ThkMin was not statistically significant in both hypoosmolar and iso-osmolar riboflavin groups (*P* > 0.5 for both).

### 3.4. Keratoconus Screening Indices

The mean SIf, KVf, Sib, and KVb values yield nonsignificant differences at 2 years postoperatively compared to their mean baseline values (*P* > 0.5 for all). However, the mean BCV values were significantly decreased from 3.37 ± 2.44 *μ*m preoperatively to 2.65 ± 1.28 *μ*m at 24 months postoperatively (*P*=0.006) (Table 4).

**Figure 2:**
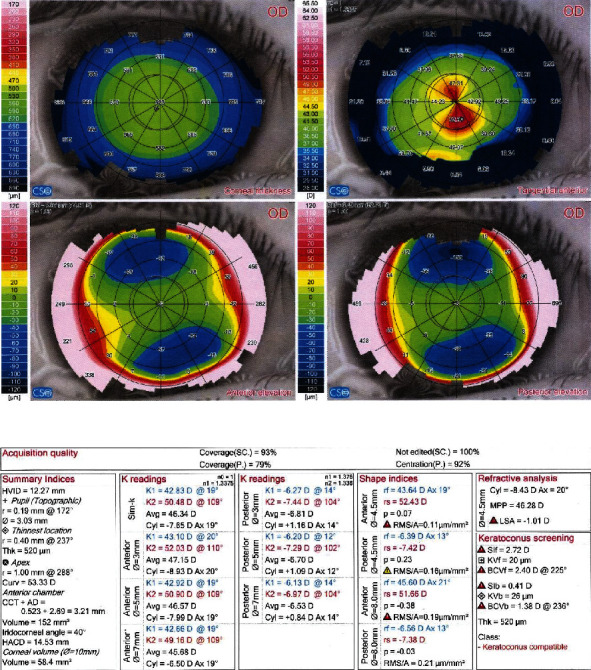
Pre-CXL (Sirius v 2.6, CSO, Italy) quad map of an 8-year male with keratoconus.

**Figure 3:**
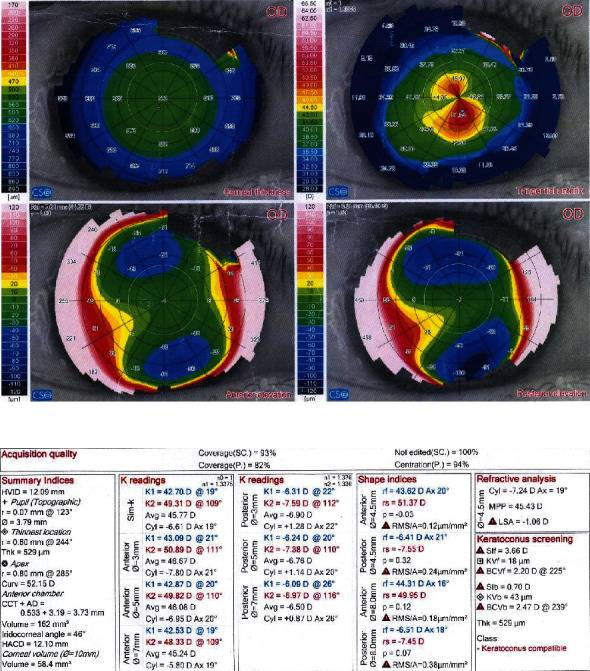
Two years post-CXL Sirius quad map of the same eye. At 2 years after treatment, the preoperative values of flat, steep, and mean keratometries decreased from 42.83 D, 50.48 D, and 46.34 D, respectively, to 42.70, 49.31, and 45.77, respectively, while the anterior maximum keratometry (curve apex) decreased from 53.33 D to 52.15 D. The minimum corneal thickness increased from 520 um to 529 um.

Figures 2 and 3 demonstrate preoperative and postoperative Sirius quad maps, respectively.

### 3.5. Safety

No cases of delayed epithelial healing or keratitis were observed within the first week. A mild nonsignificant corneal haze was observed a few days after CXL in the majority of eyes and recovered spontaneously. A single case of corneal haze was reported in one eye (3.44%); this stromal haze developed 2 months after the procedure and was resistant to the topical steroid. An increase of >1 D of the maximum keratometry (K apex) without associated loss of CDVA was reported at the 6-month visit in two (6.89%) eyes of two patients. Iso-osmolar riboflavin solution was used in these eyes as the preoperative minimum corneal thickness was more than 400 *μ*m. However, we did not retreat these eyes as visual acuity and topographic parameters were stable thereafter.

## 4. Discussion

The morphological appearance of keratoconus in children is somewhat different from that seen in adults. The ectatic cornea is more centrally located in pediatric KC [8]. In their study to evaluate the severity of KC at diagnosis, Léoni-Mesplié et al. found that affected children were more likely male, diagnosed with allergies, more frequently rubbed eyes, and had a strong family history of keratoconus [9]. Furthermore, KC in the pediatric population tends to have a more rapid and aggressive course of the disease in comparison to that of adult KC [3, 4]. This is due to the structural differences in the cornea between both age groups [4, 10]. Chatzis and Hafezi investigated the progression rate in participants with KC who were younger than 19 years, and they found that 52 (88%) out of 59 eyes exhibited evidence of progression within 12 months [3]. However, they recommended that CXL should be performed at the earliest age possible to assist in halting the disease progression rather than waiting for established progression. Corneal cross-linking improves corneal tensile strength through the combination of riboflavin and UVA light [11]. Since the advent of the original Dresden protocol, various modalities of CXL have emerged, although transepithelial CXL may be a more suitable option in pediatric KC as it is associated with less pain and fewer complications. Conversely, it has been reported that intact epithelial resistance results in suboptimal penetration of riboflavin into the corneal stroma and hence reduces the effectiveness of CXL [12]. Riboflavin also has a shielding effect to the deeper structures such as the corneal endothelium, lens, and retina by absorbing the UVA light [13]. Accelerated protocols have the advantages of reduced exposure time, better patient compliance, and reduced infection risk [14]. Several studies have demonstrated similar outcomes on the biomechanical properties of the cornea with high energy and short irradiance duration when compared to standard protocols; furthermore, the total amount of irradiance remains the most important parameter in terms of the corneal stiffening effect [15, 16]. In this study, we evaluated the visual, refractive, topographic, and tomographic outcomes of pediatric KC 24 months after accelerated CXL. To our knowledge, this is the first study reporting on this treatment method in a Syrian pediatric population of keratoconus.

Caporossi et al. reported significant flattening in both K1 and K2 at 3 years after standard CXL [17]. In contrast, our results showed that both K1 and K2 were not significantly changed after A-CXL. However, flattening of 1.2 D in the mean maximum keratometry values at 2 years after CXL was found. Consistent with our findings, Witting-Silva et al. reported that maximum keratometry flattened by approximately 1.0 D at 3 years after standard CXL [7]. Godefrooij et al. reported that 12 (22%) eyes of 54 pediatric KC eyes exhibited disease progression (increase of ≥1.0 D in the maximum keratometry) at 5 years after conventional CXL [18]. Zidan et al. evaluated the short-term outcomes of accelerated transepithelial CXL in the management of pediatric KC [19]. They reported KC progression in one eye (out of 50 eyes) at one year after treatment. The article by Iqbal et al. reported a trail that compared the standard epithelium-off CXL with accelerated epithelium-off CXL and transepithelial epithelium-on CXL in the treatment of pediatric patients with KC [20]. The researchers found that the total success rates (percentage of eyes with no deterioration of Kmax) were 100%, 94.6%, and 71.6% in the standard CXL, accelerated CXL, and transepithelial CXL groups, respectively, at month 24 after treatment. In this current study, an increase of >1 D in maximum keratometry was observed in 2 (6.89%) eyes at 6 months postoperatively. These patients were monitored at more frequent intervals, and at 24 months, the CDVA was improved in one eye and decreased one Snellen line in the other eye. At 2 years after CXL, no additional CXL or corneal transplantation was needed in any of these eyes, as there was no further deterioration in the visual acuity, corneal thickness, or topographic indices. Godefrooij et al. assumed that the decentralized cone location was the only independent underlying cause of KC progression [18]. In our study, all treated eyes had central KC (apex within 2 mm) which may explain the lower progression rate. These findings are consistent with previously reported results, which considered cone location as a major predictor of the maximum keratometry outcome [21].

Turhan et al. found that UDVA and CDVA showed no significant changes at 2 years after 9 mM/cm^2^ irradiance for 10 minutes of CXL, while CDVA was significantly improved after conventional CXL [22]. In contrast, Wise et al. found that the CDVA did not change significantly at 1 year after standard CXL [23]. Ozgurhan et al. reported their results of accelerated CXL (irradiance of 30 mW/cm^2^; 4 minutes) in pediatric patients with KC where they found that UDVA and CDVA were significantly improved, 0.13 and 0.08 logMAR, respectively, at 24 months after CXL [24]. Cinar et al. evaluated the long-term outcomes of epithelium-off A-CXL in pediatric KC where they found significant improvement in the mean UDVA and CDVA values 5 years after treatment [25]. In this study, CDVA remained unchanged or improved in 26 (89.66%) eyes and decreased 1 Snellen line in 3 (10.34%) eyes 24 months after CXL. In our previously reported study [26] that compared an accelerated epithelium-off CXL with the standard Dresden protocol, we found that 6 eyes of 36 eyes treated with the standard CXL lost two or more lines of Snellen CDVA at the final follow-op. This was explained by the significant persistent haze formation in four eyes and scar development in two eyes. In the current study, one of the eyes treated with A-CXL showed persistent haze which may explain the reduced visual acuity in this eye. However, two out of 29 eyes showed a reduction of one line in the Snellen CDVA with no evident haze or scar. We assume that the decreased visual acuity in those eyes was related to the increase in higher-order aberrations (HOAs). Zarei-Ghanavati et al. stated that increased corneal HOAs and decreased contrast sensitivity were the factors responsible for decreased visual acuity after corneal cross-linking [27]. Although our results demonstrated minimal changes in refractive and keratometric values, both UDVA and CDVA were significantly improved with mean changes of 0.13 and 0.06 logMAR, respectively. The visual improvement in pediatric KC after corneal cross-linking could be explained by the improvement in the corneal higher-order aberrations [28].

Several studies have found that corneal thickness was reduced to a greater extent after standard CXL when compared with accelerated CXL [29, 30]. Ozgurhan et al. reported that the mean minimum corneal thickness values were significantly decreased 1 month postoperatively and returned to their baseline values at 6 months after A-CXL [24]. Consistent with these findings, we observed a nonsignificant reduction (7.03 *μ*m) in the minimum corneal thickness at 24 months following treatment. Fry et al. found that hypotonic riboflavin was able to maintain corneal thickness more effectively in comparison to riboflavin/dextran CXL [31]. In our study, although nonsignificant, the hypotonic riboflavin group showed less thinning than the iso-osmolar riboflavin group at 24 months after CXL. Hafezi reported a case where CXL failed to halt the progression of KC in a very thin cornea (minimum corneal pachymetry of 268 *μ*m) despite that swelling with hypoosmolar riboflavin solution increased to 406 *μ*m and no adverse endothelial toxicity was observed [6]. However, they suggested that the preoperative minimum corneal thickness should be at least 330 *μ*m. In this study, the minimum corneal thickness was 360 *μ*m in one eye, where the preoperative Snellen UDVA improved from 0.5 to 0.6 postoperatively and the maximum anterior keratometry decreased from 53.48 D to 50.5 D 2 years postoperatively.

Ophthalmologists should be aware of intraoperative corneal pachymetry measurements. Currently, ultrasound pachymetry is the most commonly used method for intraoperative pachymetry measurements during CXL. However, using this technique is subject to limitations: only single-point measurements can be obtained, and it is necessary to have contact to obtain the measurements, thus rendering the results more prone to inter- and intraobserver variability. Furthermore, direct corneal surface contact may result in infection.

Corneal haze has commonly been reported after conventional CXL [32]. Raiskup et al. stated that advanced KC should be considered as higher risk of haze development after CXL due to low corneal thickness and high corneal curvature [33]. To some extent, this hypothesis may explain our findings; of the 29 eyes treated, one eye with preoperative apex keratometry of 64.55 D and thinnest pachymetry of 368 *μ*m showed permanent mild corneal haze following treatment. Although the haze persisted, CDVA improved 2 Snellen lines at 24 months following CXL.

Our study is not without limitations: first is its retrospective design as retrospective studies are designed to analyse preexisting data and are subject to numerous biases as a result in addition to the inferior level of evidence compared with prospective studies. Another limitation in our study is that nine of the patients had both of their eyes analyzed which could be a source of statistical bias. When both eyes are assigned to the same treatment group, less information is obtained from 2 study eyes from 1 participant than from 2 participants with 1 study eye because the participant's 2 eyes may behave similarly. Here, including the second eye provides less information about treatment effect than adding an additional participant. The small sample size and the lack of demarcation line depth measurements are also considered as other limitations of our study. Larger prospective studies are required to evaluate the impact of different CXL protocols on corneal morphology and biomechanical properties in pediatric keratoconus patients. Standardized measurements with anterior segment ocular coherence tomography (AS-OCT) in future studies may add further value in establishing demarcation line depths.

## 5. Conclusion

This study showed that accelerated CXL is a safe and effective treatment modality to stabilize pediatric keratoconus over 24 months. It also appeared to improve visual acuity in a proportion of patients. However, two (6.89%) eyes showed an increase of more than 1 D in maximum keratometry, and 3 (10.34%) eyes showed a reduction in visual acuity.

## Figures and Tables

**Table 1:** Visual and refractive outcomes after accelerated cross-linking in pediatric keratoconus.

	Preoperative					Postoperative					Mean change		*P* value
	Mean	Std. dev	Median	[95% CI]		Mean	Std. dev	Median	[95% CI]		Mean	Std. dev	
UDVA (logMAR)	0.56	0.28	0.52	0.39	0.65	0.42	0.29	0.40	0.27	0.53	0.13	0.18	**0.0003**
CDVA (logMAR)	0.34	0.23	0.3	0.20	0.40	0.28	0.22	0.22	0.12	0.32	0.06	0.13	**0.0145**
Sphere (D)	−0.50	1.40	0	−0.68	0.68	−0.10	1.01	0	−0.46	0.46	−0.40	0.79	**0.0228**
Cylinder (D)	−3.02	1.18	−3.00	−3.54	−2.46	−2.73	1.10	−2.63	−3.14	−2.11	−0.29	0.70	**0.0395**
MRSE (D)	−2.01	1.49	−1.75	−2.44	−1.06	−1.42	1.04	−1.25	−1.73	−0.77	−0.59	0.95	**0.0024**

UDVA: uncorrected distance visual acuity; CDVA: corrected distance visual acuity; D: diopter; MRSE: manifest refraction spherical equivalent; [95% CI]: 95% confidence interval. Statistically significant increased values (*P* < 0.05). Values in bold are statistically significant.

**Table 2:** Topographic and pachymetric outcomes after accelerated cross-linking in pediatric keratoconus.

	Preoperative					Postoperative					Mean change		*P* value
	Mean	Std. dev	Median	[95% CI]		Mean	Std. dev	Median	[95% CI]		Mean	Std. dev	
K1 (D)	44.92	2.20	44.46	43.43	45.49	44.74	2.46	44.29	43.15	45.43	0.19	0.77	0.1598
K2 (D)	48.71	2.05	48.84	47.90	49.78	48.77	2.78	49.1	47.82	50.38	−0.05	1.41	0.8542
K mean (D)	46.80	2.04	46.14	45.16	47.12	46.66	2.33	46.57	45.51	47.63	0.14	1.12	0.642
K apex (D)	56.97	5.24	55.62	53.15	58.09	55.84	5.37	53.2	50.44	55.96	1.13	2.00	**0.0036**
Sim cyl (D)	−3.72	1.77	−3.55	−4.36	−2.74	−4.04	2.30	−3.52	−4.59	−2.45	0.32	1.03	0.1385
ThkMin (*μ*m)	438	49	433	411	456	431	47	428	407	450	7	22	0.0836

K: keratometry; K1: flat keratometry; K2: steep keratometry; K mean: mean keratometry; K apex: apex keratometry; Sim cyl: simulated cylinder; D: diopter; ThkMin: minimum corneal thickness; *μ*m: micron; [95% CI]: 95% confidence interval. Statistically significant increased values (*P* < 0.05). The value in bold is statistically significant.

**Table 3:** Corneal thickness changes between hypoosmolar and iso-osmolar riboflavin groups

	*N*	Hypoosmolar					Iso-osmolar					*P* value
		5					24					
		Mean	Std. dev	Median	[95% CI]		Mean	Std. dev.	Median	[95% CI]		
ThkMin (*μ*m)	Preoperative	367	19	364	343	385	452	40	443	423	463	**0.0005**
	Postoperative	362	39	360	317	403	445	34	443	426	460	**0.0007**
	Mean change	5	37				7	19				0.86

	*P* value	0.5					0.0812					

*N*: number; ThkMin: minimum corneal thickness; *μ*m: micron; [95% CI]: 95% confidence interval. Statistically significant increased value (*P* < 0.05). Values in bold are statistically significant.

**Table 4:** Changes in keratoconus screening indices after accelerated cross-linking in pediatric keratoconus.

	Preoperative					Postoperative					Mean change		*P* value
	Mean	Std. dev	Median	[95% CI]		Mean	Std. dev	Median	[95% CI]		Mean	Std. dev	
SIF (D)	5.82	3.01	5.57	4.19	6.95	5.94	3.33	4.81	3.20	6.42	−0.12	1.60	0.4958
KVF (*μ*m)	28.07	12.20	27.00	21.41	32.59	26.91	14.31	26.00	19.46	32.54	1.16	4.76	0.147
SIB (D)	1.65	0.79	1.51	1.14	1.88	1.52	0.88	1.49	1.09	1.89	0.13	0.57	0.5235
KVB (*µ*m)	60.62	25.78	58.00	46.18	69.82	63.35	27.35	63.00	50.52	75.48	−2.73	15.30	0.1328
BCV (*μ*m)	3.37	2.44	2.95	1.78	4.12	2.65	1.28	2.56	1.93	3.19	0.72	2.29	**0.0068**

SIf: symmetry index front; D: diopter; KVf: keratoconus vertex front; *μ*m: micron; SIb: symmetry index back; KVb: keratoconus vertex back; BCV: Baiocchi Calossi Versaci index (total); [95% CI]: 95% confidence interval. Statistically significant increased values (*P* < 0.05). The value in bold is statistically significant.

## Data Availability

The datasets used and analyzed during the current study are available from the corresponding author (AS) upon reasonable request.
